# Low lamin A expression in lung adenocarcinoma cells from pleural effusions is a pejorative factor associated with high number of metastatic sites and poor Performance status

**DOI:** 10.1371/journal.pone.0183136

**Published:** 2017-08-14

**Authors:** Elise Kaspi, Diane Frankel, Julien Guinde, Sophie Perrin, Sophie Laroumagne, Andrée Robaglia-Schlupp, Kevin Ostacolo, Karim Harhouri, Rachid Tazi-Mezalek, Joelle Micallef, Hervé Dutau, Pascale Tomasini, Annachiara De Sandre-Giovannoli, Nicolas Lévy, Pierre Cau, Philippe Astoul, Patrice Roll

**Affiliations:** 1 Aix Marseille Univ, INSERM, GMGF, Marseille, France; 2 APHM, Hôpital la Timone, Service de Biologie Cellulaire, Marseille, France; 3 APHM, Hôpital Nord, Department of Thoracic Oncology–Pleural diseases–Interventional pulmonology, Marseille, France; 4 APHM, Hôpital la Timone, Département de Génétique Médicale et Centre de Ressources Biologiques, Marseille, France; 5 APHM, Hôpital la Timone, Service de Pharmacologie Clinique & Centre d’Investigation Clinique—CPCET, Marseille, France; 6 Aix Marseille Univ, CNRS, INT, Inst Neurosci Timone, Marseille, France; 7 Aix Marseille Univ, APHM, Marseille Early Phases Cancer Trials Center CLIP^2^, Marseille, France; 8 Aix Marseille Univ, Marseille, France; Hunter College, UNITED STATES

## Abstract

The type V intermediate filament lamins are the principal components of the nuclear matrix, including the nuclear *lamina*. Lamins are divided into A-type and B-type, which are encoded by three genes, *LMNA*, *LMNB1*, and *LMNB2*. The alternative splicing of *LMNA* produces two major A-type lamins, lamin A and lamin C. Previous studies have suggested that lamins are involved in cancer development and progression. A-type lamins have been proposed as biomarkers for cancer diagnosis, prognosis, and/or follow-up. The aim of the present study was to investigate lamins in cancer cells from metastatic pleural effusions using immunofluorescence, western blotting, and flow cytometry. In a sub-group of lung adenocarcinomas, we found reduced expression of lamin A but not of lamin C. The reduction in lamin A expression was correlated with the loss of epithelial membrane antigen (EMA)/MUC-1, an epithelial marker that is involved in the epithelial to mesenchymal transition (EMT). Finally, the lamin A expression was inversely correlated with the number of metastatic sites and the WHO Performance status, and association of pleural, bone and lung metastatic localizations was more frequent when lamin A expression was reduced. In conclusion, low lamin A but not lamin C expression in pleural metastatic cells could represent a major actor in the development of metastasis, associated with EMT and could account for a pejorative factor correlated with a poor Performance status.

## Introduction

Malignant cell identification and characterization in pleural effusions are essential for the diagnosis and management of patients affected by primary or metastatic cancer. In this context, the identification of new biomarkers is required to improve the differential diagnosis between cancer subtypes, to choose the most appropriate therapy, and to make prognostic correlations.

Nuclear abnormalities, such as aberrant shape, irregular chromatin texture, and prominent nucleoli, are hallmarks of carcinoma cells [1,2] and are commonly used to diagnose malignancies [2,3]. The nuclear matrix is thought to be a principal determinant of nuclear architecture, particularly through its interactions with the nuclear envelope [4–6]. Nuclear matrix results from chemical preparation, using high salt saline solution, and is composed of the peripheral nuclear *lamina*, an internal network of proteins and residual nucleolus. Nuclear matrix includes nucleoskeleton, mostly composed of lamin filament networks, and other proteins, such as ribonucleoproteins, nuclear mitotic apparatus (NuMA)… [7,8]

The nuclear *lamina* is a network of lamin filaments interacting with lamin-associated proteins and is located underneath the inner nuclear envelope. In both the nuclear *lamina* and matrix, lamins act as scaffolding proteins that are involved in numerous nuclear functions, such as chromatin organization, DNA repair, DNA replication, transcription, and epigenetic regulation, with regulatory effects on the cell cycle and differentiation, apoptosis, and senescence [9,10].

The type V intermediate filament lamins are the principal components of the nuclear matrix, including the nuclear *lamina*. Lamins predominate in the nuclear *lamina* and are more diffuse throughout the nucleoplasm, at significantly lower concentrations than in the nuclear *lamina* [3,8,11]. Lamins are divided into A-type and B-type, which are encoded by three genes, *LMNA*, *LMNB1*, and *LMNB2*. The alternative splicing of *LMNA*, located at chromosome 1q21.1–21.3, produces two major A-type lamins, lamin A and lamin C, which are expressed in most differentiated cells, and two minor isoforms, lamin AΔ10, which is expressed in some carcinoma cell lines [12], and lamin C2, which is specifically expressed in germ cells. B-type lamins are encoded by two distinct genes, *LMNB1*, located at chromosome 5q23, and *LMNB2*, located at chromosome 19p13 [4,12–15]. Lamins B1 and B2 show ubiquitous expression, whereas lamin B3, produced from *LMNB2* by alternative splicing, is specifically expressed in germ cells [4,9].

Lamins A, B1, and B2 are first expressed as cytosolic precursors called prelamins that undergo numerous post-translational processing steps involving their carboxy terminal CaaX box. First, a farnesyltransferase adds a farnesyl group to the cysteine. This 15-carbon hydrophobic group temporarily (prelamin A) or permanently (prelamin B; mature B-type lamins) anchors the prelamins to the cytosolic leaflet of the endoplasmic reticulum membrane or of the outer nuclear envelope. During the next step, FACE1/ZMPSTE24 or FACE2/Rce1 protease removes the last three amino acids, aaX. Then, the farnesylated cysteine is methylated by an isoprenylcysteine carboxymethyl transferase (ICMT). After this last step of their processing, mature B-type lamins remain farnesylated and carboxymethylated, are imported into the nucleoplasm, and are located in the nuclear *lamina*, anchored to the inner nuclear envelope. Unlike B-type prelamins, prelamin A undergoes another proteolytic cleavage specifically performed by FACE1/ZMPSTE24, which clips off the last 15 residues, including the farnesylated and carboxymethylated cysteine. This removal leads to a mature soluble lamin A, which is imported into the nucleoplasm and is localized in both the nuclear *lamina* and the rest of the nuclear matrix [3,4,14,16]. Lamin C, lacking the CaaX prenylation box, is directly synthesized as a mature form and shares the same nuclear location as lamin A [4,14,16]. Mutations in *LMNA* or defects in prelamin A posttranslational processing cause a wide range of diseases called laminopathies [3,4,16].

Previous studies have suggested that lamins are involved in tumor development and progression [17]. Variations in lamin localization and/or expression levels have been reported in several cancer subtypes [18,19]. The heterogeneous expression of lamin A/C has been described [3,14], primarily using western blots and immunohistochemical staining, in cancer cells obtained from colorectal [6,20–22], gut [23], gastric [6], lung [18,19], breast [1,24], prostate [9,25,26], testis [27], skin [28,29] hematologic [10], cervical [30], endometrial [31] and ovarian [32,33] tumors. Opposite results have been reported concerning the prognosis associated with a reduced or lack of expression of lamin A/C in gastric and advanced colon carcinomas [6,21–23]. In lung cancer, only a few older studies in cell lines or human tissues have shown reduced lamin A/C expression in small cell lung cancer (SCLC) in contrast with non-SCLC [17–19]. A few publications have described decreased expression of B-type lamins in gastric and colon carcinomas [23] and in some lung adenocarcinomas [18], whereas the reverse was observed in prostate carcinoma [25,26].

In addition to these quantitative abnormalities, the aberrant cytoplasmic localization of A- and B-type lamins has also been observed in some colon, gastric, and pancreatic cancer and in non-SCLC cells [17,23].

Altogether, these studies propose lamin expression as a biomarker for cancer diagnosis, prognosis, and/or follow-up [14]. However, previous studies have only analyzed human cancer cell lines and lung cancer tissues from biopsies.

The aim of the present study was to investigate lamins A, C, and B1 as potential biomarkers in cancer cells from metastatic pleural effusions.

## Materials and methods

### Study design

This study was an observational study with no intervention. Patients were recruited in the Thoracic Oncology, Pleural Disease, and Interventional Pulmonology Department at Marseille North Hospital. The study was registered on the ClinicalTrials.gov web site (identifier: NCT01284777). The protocol was approved by the Marseille Ethical Committee (Comité de Protection des Personnes Sud Méditerranée I–References number: 2010-A00295-34) and performed in accordance with the Declaration of Helsinki. Subjects provided informed written consent before participation.

### Cytological analysis

Pleural effusions were received in the Cell Biology Laboratory at La Timone Hospital of Marseille for conventional cytological diagnosis. Briefly, a fraction of the fresh sample was cytospun (Thermo Electron Corporation, Cheshire, United Kingdom) at 450 g for 3 minutes, and another portion was smeared from the sediment after centrifugation (Jouan, Saint-Herblain, France) at 600 g for 5 minutes.

The slides were air-dried or fixed in acetone and methanol and then stained with May-Grunwald-Giemsa (MGG) or Papanicolaou stains, respectively. The remaining fraction of the sample was used to prepare slides and cell pellets and was stored at -70°C until use.

Using a light microscope (Leica, Wetzlar, Germany), malignant cells were manually counted, and conventional cytological analysis was completed by immunocytochemical phenotyping. Histological confirmation was achieved in most cases (90%) by conventional analysis and immunohistochemistry on paraffin sections. For the other patients, biopsy and pathological examination could not be performed. Therefore, histologic subtype and primitive tumor localization were defined based on the cytology only associated with the physical examination and radiological imaging according to international recommendations [34].

### Primary antibodies used for immunofluorescence (IF) and/or western blotting (WB)

The following primary antibodies were used for immunofluorescence (IF) and/or western blotting (WB): mouse anti-lamin A [ab8980, Abcam, Paris, France (IF: 1/100, WB: 1/200)]; rabbit anti-lamin C [BP4505, Acris, Herford, Germany (IF: 1/100, WB: 1/230)]; mouse anti-lamin A/C [MAB3211, Merck Millipore, Massachusetts, United States (WB: 1/200)]; goat anti-lamin A/C [sc6215, Santa Cruz, Texas, United States (WB: 1/200)]; rabbit anti-lamin A/C [sc20681, Santa Cruz (WB: 1/200)]; rabbit anti-lamin B1 [ab16048, Abcam (WB: 1/250)]; glyceraldehyde 3-phosphate dehydrogenase (GAPDH) [MAB374, Merck Millipore (WB: 1/40,000)].

For IF, the following isotype antibodies were used as negative controls: mouse IgG (015-000-003, Jackson ImmunoResearch, Suffolk, UK); and rabbit IgG (AB-105-C, R&D, Minnesota, United States).

### Dermal fibroblast culture and total protein extraction

The culture and storage of control dermal fibroblasts were performed by the Biological Resource Center of the Department of Medical Genetics (Marseille Timone Hospital).

Cells were cultured in DMEM (Life Technologies) containing 20% FBS (Life Technologies), 2 mM L-glutamine (Life Technologies), and 2X penicillin-streptomycin (Life Technologies) at 37°C in a humidified atmosphere containing 5% CO_2_. Total fibroblast proteins were extracted (1% Triton X100, 0.1% SDS, 0.5% sodium deoxycholate, 150 mM NaCl, 1 mM EDTA, 20 mM Tris-HCl pH 7.5, 1X Complete EDTA-free protease inhibitor cocktail, 1 mM Na_3_VO_4_, 1 mM PMSF). Cells were sonicated twice (30 seconds each), incubated at 4°C for 30 minutes, and then centrifuged at 10,000 g for 10 minutes. Protein concentrations were determined with the BCA™ Protein Assay (Thermo Scientific) according to the manufacturer’s instructions [35]. Dermal fibroblasts were used as controls in western blot experiments.

### Nuclear protein extraction from pleural effusion cells

Nuclear proteins were extracted from frozen cell pellets [36]. Briefly, cells were incubated during centrifugation (10 min, 800 g, 4°C) in lysis buffer #1 (10 mM Tris HCl pH 7.5, 30 mM NaCl, 3 mM MgCl_2_, 1 mM PMSF, 0.5 μg/ml aprotinin, 0.5% NP40, and 1X Complete EDTA-free protease inhibitor cocktail (Roche, Meylan, France)).

The supernatant was then removed, and the pellet was washed and incubated for 1 h at 4°C in lysis buffer #2 (50 mM Tris HCl pH 7.5, 250 mM sucrose, 5 mM MgSO_4_, 1 mM PMSF, 0.5 μg/ml aprotinin, 0.5% NP40, 500 kU/ml DNase (D-5025, Sigma), 25 kU/ml RNase (D-5503, Sigma), and 1X Complete EDTA-free protease inhibitor cocktail). After centrifugation (800 g, 15 minutes, 4°C), the supernatant was removed, and the pellet was dissolved in 1.6 M NaCl. Protein concentrations were determined using the BCA™ Protein Assay (Thermo Scientific, Courtaboeuf, France) according to the manufacturer’s instructions.

### Western blotting and lamin quantification

Proteins were extracted and analyzed by a standard western blotting procedure, described as follows.

Protein lysates were separated on Criterion™ XT 7% Tris-Acetate precast gels (345–0135, Bio-Rad, California, United States) and transferred to Immobilon-FL PVDF membranes (Merck-Millipore). Membranes were blocked for one hour in 1:2 blocking buffer for near-infrared fluorescent western blotting (Rockland, Pennsylvania, United States). The blocked membranes were incubated with primary antibodies for one hour at RT and then washed and incubated with IR-Dye-conjugated secondary antibodies for one hour at RT. Bound antibodies were detected and analyzed on an Odyssey^®^ V3.0 imaging system (LI-COR Biosciences, Homburg, Germany) according to the manufacturer’s instructions.

Secondary antibodies conjugated with IR-Dye 800 or 680 were used according to the manufacturer’s instructions (926–32212, 926–32214, 926–32223, 926–32224, 1/5000, LI-COR^®^ Biosciences) [37].

The quantities of lamin A, C, and B1 proteins were measured by fluorescence at infrared wavelengths. Lamin A expression was normalized to the total amount of lamins A and C, and a ratio was established [Lamin A / (Lamin A + Lamin C)].

### Immunofluorescence microscopy

Cells were centrifuged (450 g, 5 minutes) in a Cytospin (Thermo Electron Corporation), and slides were stored at -70°C prior to use. Cells were immunostained using a previously described protocol (37). Briefly, after 15 minutes of fixation at room temperature (RT) in a 4% paraformaldehyde + 2% sucrose solution, cells were permeabilized using a permeabilization buffer (0.5% Triton X-100, 50 mM NaCl, 300 mM sucrose, 20 mM HEPES pH 7.5, 3 mM MgCl_2_) for 3 minutes at RT. The cells were then incubated with the primary antibodies for 40 minutes at 37°C. After washing, the cells were incubated with secondary antibodies (A11001, A11055, A11012; Life Technologies, Saint-Aubin, France; 1/400) for 20 minutes at 37°C. The nuclei were counterstained with DAPI (0.1 **μ**g/mL, Sigma, Lyon, France) for 10 minutes at RT. The slides were mounted using FluorSave™ reagent (Merck Millipore) and were observed on a fluorescence microscope (ApoTome.2 Zeiss).

### Flow cytometry

A flow cytometry analysis was performed on adenocarcinoma cells from seven patients, on the remaining available samples.

Cells were stored in medium containing 90% FBS and 10% DMSO at -70°C until use. Red blood cells were lysed before storage using NH_4_Cl lysis buffer. Cells were fixed at RT in 4% paraformaldehyde solution and washed in PBS containing 0.2 M glycine prior to permeabilization at RT using the homemade permeabilization buffer detailed in the immunofluorescence method. Cells were then incubated in PBS containing 2% BSA for 20 minutes and for one hour with the following antibodies: anti-CD45-PECy7 (MHCD4512; Life Technologies), anti-EMA-PE (355604; Biolegend, California, United States), and anti-lamin A-AF647 (Ab8980; Abcam); the anti-lamin A antibody was labeled with the APEX antibody labeling kit Alexa 647 (A10475; Life Technologies). The LIVE/DEAD^®^ Fixable Near-IR Dead Cell Stain Kit (L10119, Life Technologies) was used to determine the viability of cells.

Lamin A and EMA (epithelial membrane antigen, also known as MUC-1) protein expression was analyzed in malignant cells by flow cytometry (Attune^®^, Life Technologies). The percentage of positive cells, median fluorescence intensity, and standard deviation (MFI ± SD) were measured in an average of 14,000 live, CD45-negative cells that were considered carcinoma cells. Anti-IgG1k-PE (400114, Biolegend), anti-IgG1-PeCy7 (MG112, Life Technologies), and anti-IgG3-AF647 (560803, BD Pharmingen, Le Pont de Claix, France) were used as isotype controls.

### Statistical analyses

The statistical analysis was performed with the Prism software (GraphPad Software Inc., California, United States). Significant differences were determined using t-test or Mann-Whitney test; Chi-square or Fisher tests were conducted to evaluate the differences between groups for qualitative variables; and correlations were established using Pearson or Spearman tests, depending on the Gaussian distribution of the values.

All statistical results were 2-sided and a *P-*value < 0.05 was considered significant.

## Results

### Participant characteristics

Patients diagnosed with a metastatic pleural effusion from lung adenocarcinoma in our institution were prospectively recruited. 32 patients were enrolled because their pleural effusion contained at least 20% adenocarcinoma cells.

Patient demographics and clinical and cytological data were prospectively collected, at the time of the pleural puncture (Table 1). The cohort included 14 females and 18 males, with a median age of 69 years (range 50–96 years).

**Table 1:** Pathological and clinical characteristics of patients, according lamin A expression.

Clinicopathological variables Number of patients (%)	Low (n = 12)	High (n = 20)	p-value
**Gender**			1
- Male	7 (58%)	11 (55%)	
- Female	5 (42%)	9 (45%)	
**Age, years (median)**	66 (50;96)	69 (50;90)	0.95
**Smoking history**	8 (67%)	16 (80%)	0.4
**Professional exposure**	3 (25%)	3 (15%)	0.65
**Mutation**			0.68
- *EGFR*	2 (17%)	4 (20%)	
- *KRAS*	2 (17%)	3 (15%)	
- *BRAF*	1 (8%)	0	
- *ALK* or *ROS1* rearranged	0	1 (5%)	
- Wild type or unknown	7 (58%)	12 (60%)	
**WHO Performance Status** (median) Patients repartition:	2	1	**0.007**
- 0	1 (8%)	10 (50%)	**0.037**
- 1	3 (25%)	5 (25%)	
- 2	6 (50%)	5 (25%)	
- 3	2 (17%)	0	
- 4	0	0	
**Association of bone, lung and pleural metastasis**	9 (75%)	5 (25%)	**0.01**

### Reduced expression of lamin A but not of lamin C was observed in a sub-group of lung adenocarcinomas

Lamin A, B1, and C bands were observed by western blot at the expected sizes (72 kDa, 68 kDa, and 65 kDa, respectively).

**Fig 1:**
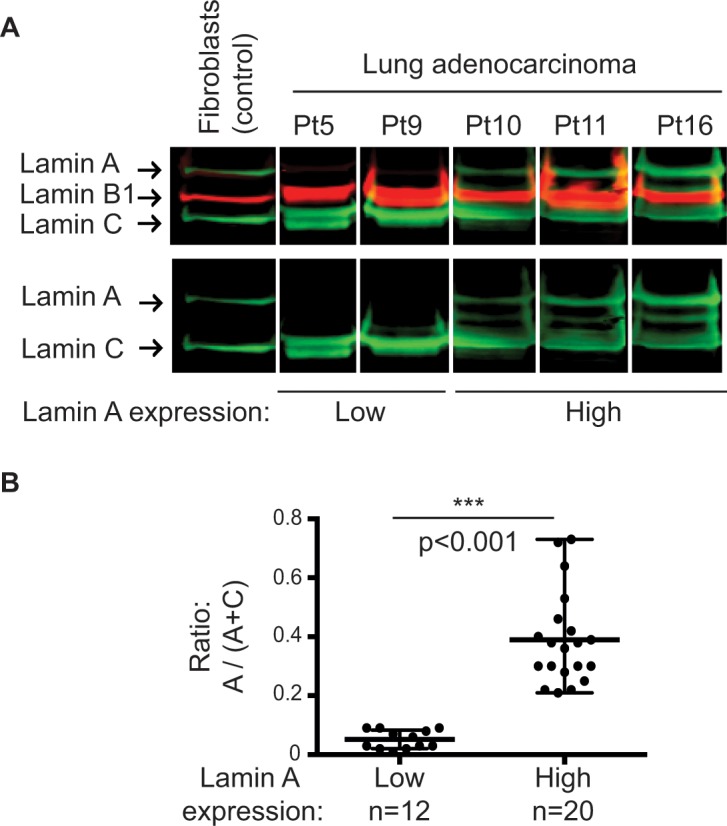
Expression pattern of lamins in lung adenocarcinoma cells from metastatic pleural effusions. (A) Representative results of western blot analysis of nuclear extracts from patients (Pt) 5, 9, 10, 11, and 16 and from total proteins extracts of control dermal fibroblasts using a mouse anti-lamin A/C antibody (Jol2). (B) Analyses of lamin A levels quantified after normalization with the total amount of lamins A and C showed two lamin A expression groups (p<0.001; Mann–Whitney test).

A strong reduction in lamin A but not in lamin C expression was specifically observed in 12 (37.5%) patients with lung adenocarcinoma pleural metastasis using a mouse anti-lamin A/C antibody (Jol2) (Panel A in Fig 1 and S1 Fig). This result was confirmed by using two others anti-lamin A/C antibodies from goat or rabbit, as well as anti-lamin A and anti-lamin C specific antibodies (Panel A in S2 Fig). Moreover, same results were obtained using total proteins extraction (Panel B in S2 Fig).

The lamin ratio [r = A / (A + C)] distinguished two groups of lung adenocarcinoma patients; 37.5% (12/32) of patients are in the low lamin A expression group with a ratio significantly reduced (p<0.001, Mann-Whitney test), exhibiting an eightfold decrease (r = 0.05 ± 0.03) compared to the patients in the high lamin A expression group (r = 0.39 ± 0.15) (Panel B in Fig 1).

**Fig 2:**
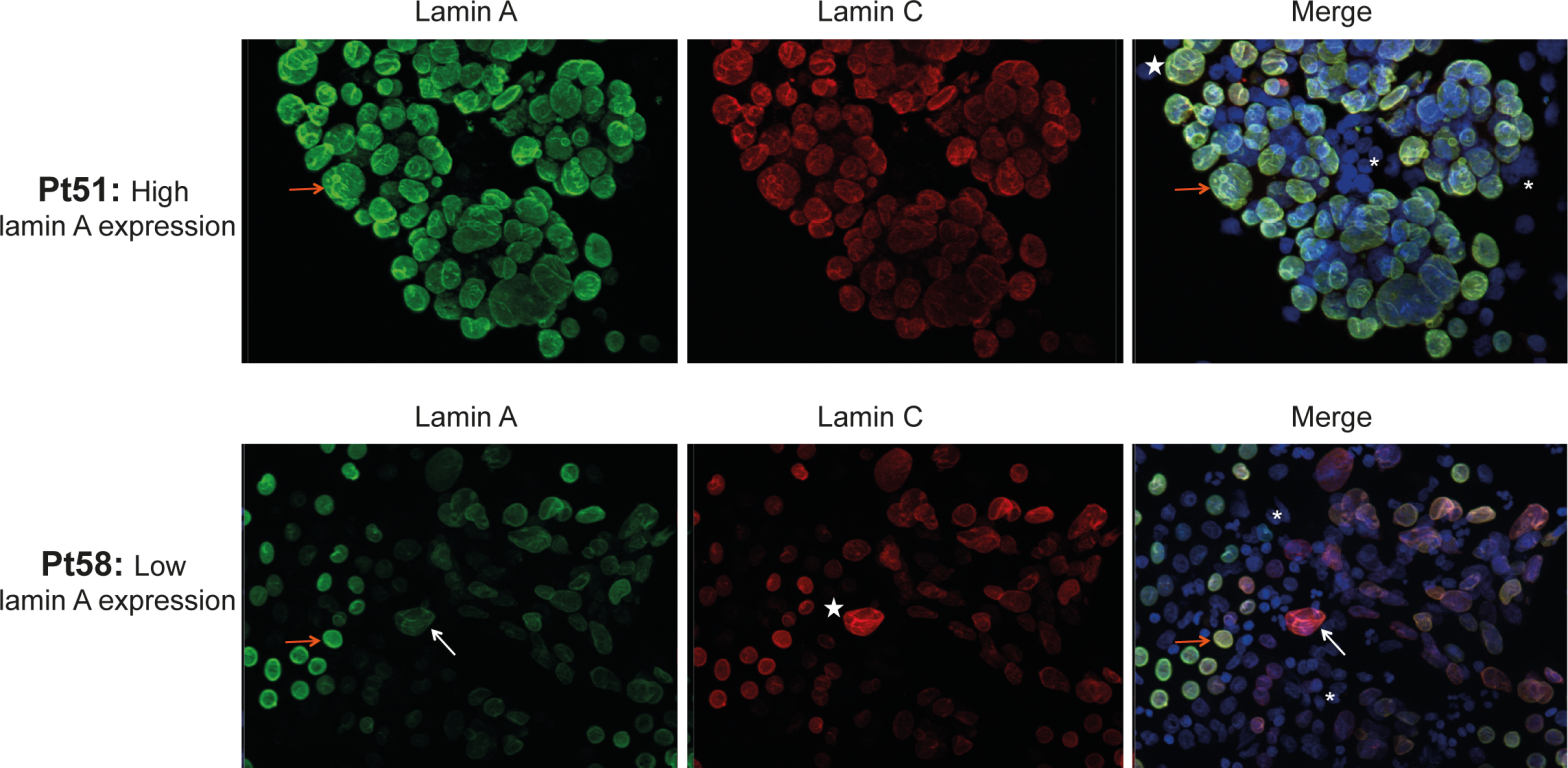
Immunofluorescence staining of lamins A and C in cells from metastatic pleural effusions. Cells from metastatic pleural effusions of lung adenocarcinoma from high (Patient (Pt) 51) and low (Pt 58) lamin A expression group were stained with lamin A and lamin C antibodies. DAPI: counterstained nuclei. White stars: dyskaryotic nuclei of cancer cells. Orange arrows: cancer cells with a high lamin A expression. White arrows: cancer cells with a low lamin A expression. Asterisk: normal leukocytes.

In accordance with Western blot analysis, immunofluorescence experiments showed, in cancer cells, a variable expression of lamin A depending on patient group (low or high lamin A expression group), whereas positive nuclear staining with anti-lamin C antibody was consistently observed. As shown in Fig 2, most adenocarcinoma cells from patients from high lamin A expression group were intensively positive for lamin A antibody staining (ex: Pt51), whereas a high part of adenocarcinoma cells from patients from low lamin A expression group showed a weak staining (ex: Pt58).

In normal leukocytes, lamin A/C expression was very weak or absent. Cytological analysis showed that cancer cells exhibited nuclear morphological abnormalities, such as dyskaryosis, blebs, and micronuclei. No aberrant cytoplasmic localization of lamin A/C was observed (Fig 2).

### Reduced lamin A expression was correlated with the loss of EMA/MUC-1 in adenocarcinoma cells

**Fig 3:**
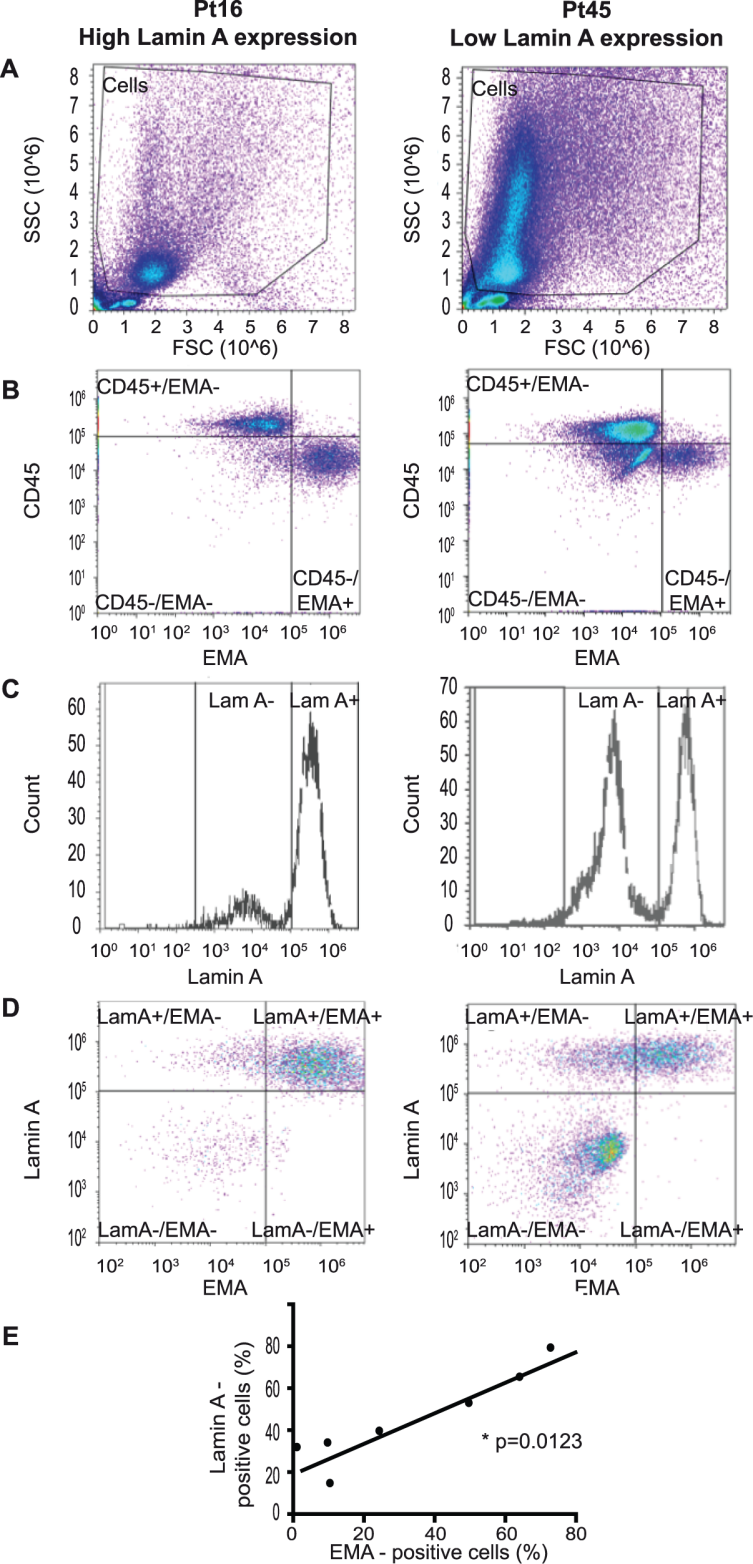
Flow cytometry analysis of lamin A and EMA in adenocarcinoma cells. (A to E) Representative results of lamin A expression in malignant cells contained in 2 metastatic pleural effusions from lung adenocarcinoma (left panel = Patient (Pt) 16 and right panel = Pt 45) using flow cytometry. Positivity thresholds were defined using isotype controls. **Cell analysis strategy:** (A) Cells were selected using SSC and FSC criteria. (B) Live cells lacking CD45 expression were considered adenocarcinoma cells (CD45-/EMA- and CD45-/EMA+ cells). Leukocytes (CD45+/EMA- cells) were excluded from analysis. (C and D) **Lamin A and EMA expression in adenocarcinoma cells.** See also Table 2.(E) **Correlation of EMA and lamin A expression in adenocarcinoma cells:** The percentage of lamin A-expressing adenocarcinoma cells was positively correlated with the percentage of EMA-expressing cells (p = 0.0123; Spearman test).

Flow cytometry analysis was performed on adenocarcinoma cells from five patients in the high lamin A expression group (patients 16, 21, 34, 37 and 38) and two patients in the low lamin A expression group (patients 20 and 45). Dead cells and leukocytes were excluded from the analysis by positive staining with the LIVE/DEAD^®^ viability marker and CD45, respectively. CD45-negative cells were considered adenocarcinoma cells (Panels A and B in Fig 3), as normal mesothelial cells accounted for less than 2% of these pleural effusions. Lamin A and EMA (epithelial membrane antigen, also known as MUC-1) protein expression was then specifically studied in these adenocarcinoma cells (Panels C and D in Fig 3). Three main adenocarcinoma cell populations were defined: cells expressing both lamin A and EMA (LamA+/EMA+); cells lacking both lamin A and EMA (LamA-/EMA-); and cells expressing lamin A associated with the loss of EMA (LamA+/EMA-). The percentages of these three main cell populations differed between patients (Panel D in Fig 3 and Table 2). A fourth minor cell population expressing EMA but lacking lamin A (LamA-/EMA+) was considered negligible because it represented less than 4% of cells.

**Table 2:** Lamin A and EMA expression in adenocarcinoma cells.

Patient	Pt16		Pt45	
	%	MFI	%	MFI
**Lamin A- cells**	18.7	8,068	54.8	6,202
**Lamin A+ cells**	79.3	352,369	39.7	574,321
**EMA- cells** (EMA-/LamA- and EMA-/LamA+ cells)	27.2	24,210	75.6	21,542
**EMA+ cells** (EMA+/LamA- and EMA+/LamA+ cells)	72.8	835,349	24.3	393,542

LamA = lamin A; MFI = median fluorescence intensity.

All patients exhibited an adenocarcinoma cell population completely lacking lamin A, but the percentage of this cell population differed between patients, depending on patient group (low or high lamin A expression group) (Panels C and D in Fig 3 and Table 2). For example, as shown in Table 2, 18.7% of adenocarcinoma cells from Patient 16 (Pt16, from high lamin A expression group) were negative for lamin A staining whereas 54.8% of adenocarcinoma cells from Patient 45 (Pt45, from low lamin A expression group) lacked lamin A expression. Thus, these results illustrate that in patients from low lamin A expression group (ex: Pt45), adenocarcinoma cell population completely lacking lamin A is predominant compared to patients from high lamin A expression group (ex: Pt16) (18.7% and 54.8%, respectively).

Moreover, the expression marker for carcinoma cell differentiation, EMA, was also lacking in some adenocarcinoma cells (Panel D in Fig 3), and interestingly, the percentage of lamin A+ adenocarcinoma cells was positively correlated with the percentage of EMA+ cells (p = 0.0123) (Panel E in Fig 3).

These flow cytometry experiments identified a sub-population of metastatic lung adenocarcinoma cells lacking lamin A expression, and we showed that this decrease in lamin A expression was correlated with the loss of EMA/MUC-1.

### Lamin A expression in lung adenocarcinoma cells was inversely correlated with the number of metastatic sites and the WHO Performance status

Clinical data from the 32 patients with lung adenocarcinoma were collected at the time of the pleural puncture and analyzed according to their high or low lamin A expression status (Table 1).

**Fig 4:**
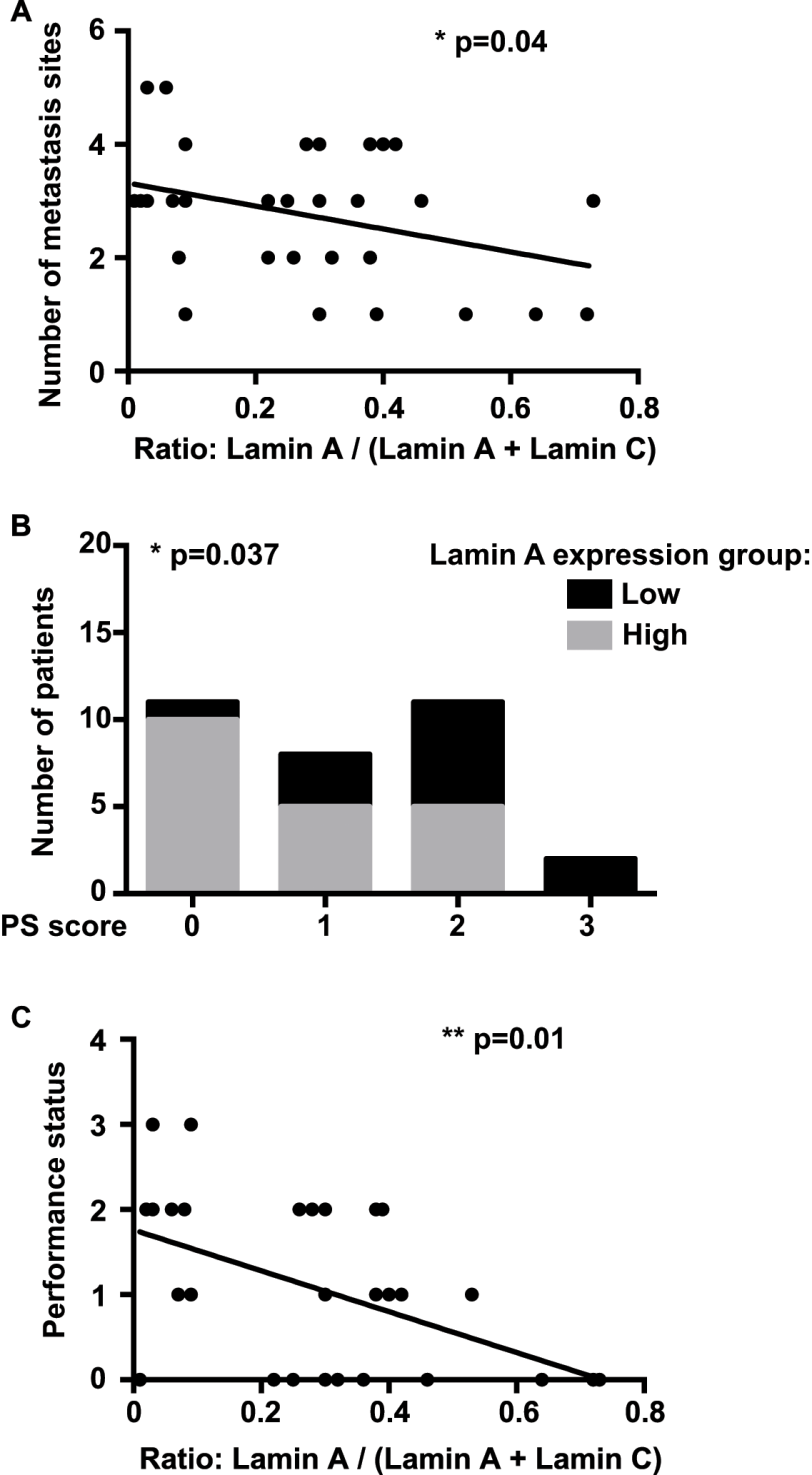
Correlation between Lamin A expression in metastatic lung adenocarcinoma cells and number of metastatic sites and WHO Performance status. (A) The number of metastasis sites was inversely correlated with lamin A expression (p = 0.04; Pearson test). (B) The patients repartition was statistically different according the lamin A expression group (p = 0.037; Chi-square test). PS = Performance status. (C) The WHO Performance status was inversely correlated with lamin A expression (p = 0.01; Spearman test).

Interestingly, the number of total metastatic sites was inversely correlated with the lamin ratio [A / (A + C)], reflecting lamin A expression (p = 0.04; Pearson test) (Panel A in Fig 4). The association of bone, lung and pleural metastasis was more frequently observed in patients of the low lamin A expression group (9/12; 75%) compared with the patients belonging to the high lamin A expression group (5/20; 25%) (p = 0.01; Fisher test).

Moreover, patients from the low lamin A group exhibited a higher Performance status score than those from the high lamin A group (2 *vs* 1, respectively; *p* = 0.007, unpaired t-test) and the patients repartition was statistically different according the lamin A expression group (*p* = 0.037; Chi-square test) (Panel B in Fig 4). In addition, the Performance status score was inversely correlated with the lamin ratio [A / (A + C)] (*p* = 0.01; Spearman test) (Panel C in Fig 4).

## Discussion

We combined immunofluorescence, western blotting, and flow cytometry for the first time to study lamins in cancer cells from metastatic pleural effusions. Previous studies have already described lamin expression abnormalities in cancer cells but have been restricted to cancerous lung tissues from primitive tumor biopsies and have relied primarily on immunohistochemical methods [17–19].

Interestingly, we reported reduced expression of lamin A but not of lamin C in a sub-group of metastatic lung adenocarcinoma patients. No variations in lamin B1 expression were observed in these cells.

Lamins A and C, which are encoded by the same *LMNA* gene, both result from alternative splicing in exon 10, which produces two different mRNAs that encode prelamin A (the precursor of lamin A) and lamin C [4]. Therefore, we hypothesize that the loss of lamin A but not lamin C expression in pleural metastatic adenocarcinoma cells could result from regulation by miRNAs or splicing factors.

Recent publications reported that miR-9 negatively controls lamin A but not lamin C expression due to the specific targeting of lamin A mRNA in neural cells [38,39]. It would be thus very interesting to investigate miR-9 expression using RT-qPCR assays in metastatic pleural effusions from lung adenocarcinoma in the two lamin A expression groups. As these samples contained cancer cells mixed with miR-9 expressing leukocytes, it would be useful to correlate the level of miR-9 with lamin expression specifically in metastatic cells after their isolation by FACS selection or using magnetic beads.

The splicing factors involved in the alternative production of lamins A and C are still unknown. The serine-arginine (SR)-rich splicing factor 1 (SRSF1) controls *LMNA* pre-mRNA alternative splicing [40], leading to the production of progerin (the truncated prelamin A protein produced in HGPS (Hutchinson-Gilford Progeria Syndrome) cells and during physiological aging) [4,41]. SRSF1 has been proposed as a proto-oncogene because it is overexpressed in many cancers [42]. In particular, in lung adenocarcinoma, SRSF1 overexpression is associated with a more aggressive phenotype, the presence of metastases, and chemotherapy resistance [43]. Progerin was found to be expressed in many human cancer cell lines, and its ectopic expression in a prostate cancer cell line led to enhanced growth and stronger tumorigenic potential *in vivo* after subcutaneous injection into nude mice [44].

Our flow cytometry analyses identified a sub-population of metastatic lung adenocarcinoma cells lacking lamin A expression, and we showed that this decrease in lamin A expression was correlated with the loss of EMA/MUC-1.

The epithelial to mesenchymal transition (EMT) is characterized by the downregulation of epithelial differentiation markers, such as EMA, and is associated with the upregulation of mesenchymal markers [45,46]. EMT increases cell motility and invasiveness and is correlated with invasive tumor metastasis and poor prognosis [43,46–48]. Nevertheless, several publications have described EMA as an EMT inducer due to glycosylation and overexpression in carcinomas [47,48]. In future studies, it would be useful to compare both lung adenocarcinoma metastatic cells and primary tumors to analyze EMA and lamin A expression, together with other EMT markers that are already known to evolve during tumor progression or metastasis.

Some studies have reported SRSF1 and miR-9 involvement in EMT during lung cancer [43,49]. Metastasis-associated lung adenocarcinoma transcript 1 (MALAT-1), a long, non-coding nuclear RNA, is known as a poor prognostic marker and a predictive marker for metastasis development in lung cancer; it targets genes involved in cell migration, tumor growth and the EMT [50–53]. Interestingly, miR-9 negatively regulates the expression of MALAT-1 [54], and MALAT-1 is implicated in the regulation of SRSF1 [50,55,56].

Therefore, it would be of interest to explore and concomitantly correlate miR-9, SRSF1 and MALAT-1 expression in lung adenocarcinoma metastatic cells and in primary tumors, and also to correlate their levels with EMT markers and lamin A expression. Furthermore, as MALAT-1 is detectable in peripheral human blood and has been proposed as a blood biomarker of NSCLC [57], we could investigate this new parameter in a prospective cohort.

Moreover, our work showed that the lamin ratio, reflecting lamin A expression, was inversely correlated with the number of total metastatic sites, with a frequent association of lung, pleural and bone metastasis in patients with low lamin A expression. Our data are consistent with a recent publication that described a reduction of lamin A but not of lamin C expression in primitive epithelial ovarian cancer tissues associated with metastasis potential and poor prognosis [32]. Similarly, in prostate carcinoma cells, reduced amounts of lamin A were correlated to an increased risk for lymph node metastasis [26]. Furthermore, the number of metastatic sites has already been linked to overall survival in lung adenocarcinoma [58,59]. Interestingly Lee et *al*., also demonstrated that bone is one of the most frequent metastasis localization in lung adenocarcinoma [58,59] whereas the presence of bone metastases is considered as an adverse prognostic factor associated with shorter survival [60].

Furthermore, miR-9 seems to be involved in tumorigenesis and cancer progression. It can act as an oncogene or a tumor suppressor; several publications described various miR-9 expression patterns between cancerous and adjacent normal tissues or depending on the tumor type [49,61–63]. Few publications have suggested that miR-9 promotes the metastasis of non-small cell lung cancer (NSCLC), and its up-regulation has been associated with poor prognosis [62,63].

However, epigenetic miR-9 silencing was described as a poor prognosis in NSCLC primary tumors, especially in squamous cell carcinoma, and as a predictive marker of lymph node metastasis by cancer cell lines derived from other tumor types [61,62].

Finally, our work showed that the reduction of lamin A expression was correlated with a poor WHO Performance status, which is considered as a gold standard prognostic measure [64]. It is proposed as the most established factor for assessing prognosis and is advised in guidelines for lung cancer treatment [64,65]. Indeed, the Performance status represents a major criteria for treatment decision in advanced lung cancer as patients with a poor Performance status are expected to be unfit for conventional treatments [64–66].

Furthermore, the Performance status has been closely linked to overall survival in advanced lung adenocarcinoma, as a poor Performance status was associated with a reduced survival [58,59,64].

These publications emphasize here the probable link of a low lamin A expression with a poor prognosis and a shorter survival outcome, which need to be confirmed in a further study.

## Conclusions

In conclusion, our work, which is a pilot study, already showed significant results, suggesting that low lamin A but not lamin C expression in pleural metastatic cells could represent a major actor in the development of metastasis, associated with EMT and could account for a pejorative factor correlated with a poor Performance status.

These results should lead to better understand at the mechanistic level, the metastasis development in which nuclear matrix, and lamins in particular, could have a central role. Moreover, we intend to confirmed our results and to analyze EMT markers in a new prospective large study to emphasize the loss of lamin A as a poor prognostic marker and to understand the link between a lack of lamin A in lung adenocarcinoma cells and their metastatic potential.

## Supporting information

S1 Fig Expression patterns of lamins A and C in metastatic lung adenocarcinoma cells from pleural effusions observed by western blot analysis. Representative results from various experiments using total protein extracts of dermal fibroblasts (control) and extracts of cells contained in metastatic pleural effusions from the 32 patients (Pt). Patients were separated according to the ratio A/(A+C). Patients with a ratio higher than 0.1 belonged to the “High lamin A expression group” whereas patients with a ratio under 0.1 were classified in the “Low lamin A expression group”.
(TIF)

S2 Fig Confirmation of lamin A expression pattern in lung adenocarcinoma cells by western blot analysis, using different antibodies and total proteins extraction. (A) Representative results of western blot analysis of nuclear extracts from patients (Pt) 5, 9, 10, 11, 16, 20 and 31 and from control dermal fibroblasts using a mouse anti-lamin A/C antibody (Jol2, MAB3211), a goat-anti-lamin A/C antibody (N18, sc6215), a rabbit-anti-lamin A/C antibody (H110, sc20681), anti-lamin A antibody (ab8980) and anti-lamin C antibody (BP4505).
(B) Representative results of western blot analysis of total protein extracts from patients (Pt) 20, 31, 45 (low lamin A group) and Pt 10, 11, 16, 21 and 27 (from high lamin A group) using a mouse anti-lamin A/C antibody (Jol2).
(TIF)

## References

[1] Capo-chichi CD, Cai KQ, Smedberg J, Ganjei-Azar P, Godwin AK, Xu X-X. Loss of A-type lamin expression compromises nuclear envelope integrity in breast cancer. Chin J Cancer. 2011 6;30(6):415–25. doi: 10.5732/cjc.010.10566 21627864 10.5732/cjc.010.10566 PMC3941915

[2] Zink D, Fischer AH, Nickerson JA. Nuclear structure in cancer cells. Nat Rev Cancer. 2004 9;4(9):677–87. doi: 10.1038/nrc1430 15343274 10.1038/nrc1430

[3] Butin-Israeli V, Adam SA, Goldman AE, Goldman RD. Nuclear lamin functions and disease. Trends Genet TIG. 2012 9;28(9):464–71. doi: 10.1016/j.tig.2012.06.001 22795640 10.1016/j.tig.2012.06.001 PMC3633455

[4] Cau P, Navarro C, Harhouri K, Roll P, Sigaudy S, Kaspi E, et al Nuclear matrix, nuclear envelope and premature aging syndromes in a translational research perspective. Semin Cell Dev Biol. 2014 3 28; 10.1016/j.semcdb.2014.03.021 24662892

[5] Hatch EM, Fischer AH, Deerinck TJ, Hetzer MW. Catastrophic nuclear envelope collapse in cancer cell micronuclei. Cell. 2013 7 3;154(1):47–60. doi: 10.1016/j.cell.2013.06.007 23827674 10.1016/j.cell.2013.06.007 PMC3749778

[6] Wu Z, Wu L, Weng D, Xu D, Geng J, Zhao F. Reduced expression of lamin A/C correlates with poor histological differentiation and prognosis in primary gastric carcinoma. J Exp Clin Cancer Res CR. 2009;28:8 doi: 10.1186/1756-9966-28-8 19144202 10.1186/1756-9966-28-8 PMC2632624

[7] Simon DN, Wilson KL. The nucleoskeleton as a genome-associated dynamic “network of networks.” Nat Rev Mol Cell Biol. 2011 11;12(11):695–708. doi: 10.1038/nrm3207 21971041 10.1038/nrm3207

[8] Linnemann AK, Krawetz SA. Maintenance of a functional higher order chromatin structure: The role of the nuclear matrix in normal and disease states. Gene Ther Mol Biol. 2009;13(1):231–43. 20948980 PMC2952954

[9] Kong L, Schäfer G, Bu H, Zhang Y, Zhang Y, Klocker H. Lamin A/C protein is overexpressed in tissue-invading prostate cancer and promotes prostate cancer cell growth, migration and invasion through the PI3K/AKT/PTEN pathway. Carcinogenesis. 2012 4;33(4):751–9. doi: 10.1093/carcin/bgs022 22301279 10.1093/carcin/bgs022

[10] Prokocimer M, Margalit A, Gruenbaum Y. The nuclear lamina and its proposed roles in tumorigenesis: projection on the hematologic malignancies and future targeted therapy. J Struct Biol. 2006 8;155(2):351–60. doi: 10.1016/j.jsb.2006.02.016 16697219 10.1016/j.jsb.2006.02.016

[11] Goldman RD, Gruenbaum Y, Moir RD, Shumaker DK, Spann TP. Nuclear lamins: building blocks of nuclear architecture. Genes Dev. 2002 3 1;16(5):533–47. doi: 10.1101/gad.960502 11877373 10.1101/gad.960502

[12] Machiels BM, Zorenc AH, Endert JM, Kuijpers HJ, van Eys GJ, Ramaekers FC, et al An alternative splicing product of the lamin A/C gene lacks exon 10. J Biol Chem. 1996 4 19;271(16):9249–53. 8621584 10.1074/jbc.271.16.9249

[13] Biamonti G, Giacca M, Perini G, Contreas G, Zentilin L, Weighardt F, et al The gene for a novel human lamin maps at a highly transcribed locus of chromosome 19 which replicates at the onset of S-phase. Mol Cell Biol. 1992 8;12(8):3499–506. 1630457 10.1128/mcb.12.8.3499-3506.1992 PMC364599

[14] Prokocimer M, Davidovich M, Nissim-Rafinia M, Wiesel-Motiuk N, Bar DZ, Barkan R, et al Nuclear lamins: key regulators of nuclear structure and activities. J Cell Mol Med. 2009 6;13(6):1059–85. doi: 10.1111/j.1582-4934.2008.00676.x 19210577 10.1111/j.1582-4934.2008.00676.x PMC4496104

[15] Wydner KL, McNeil JA, Lin F, Worman HJ, Lawrence JB. Chromosomal assignment of human nuclear envelope protein genes LMNA, LMNB1, and LBR by fluorescence in situ hybridization. Genomics. 1996 3 15;32(3):474–8. doi: 10.1006/geno.1996.0146 8838815 10.1006/geno.1996.0146

[16] Broers JLV, Ramaekers FCS, Bonne G, Yaou RB, Hutchison CJ. Nuclear lamins: laminopathies and their role in premature ageing. Physiol Rev. 2006 7;86(3):967–1008. doi: 10.1152/physrev.00047.2005 16816143 10.1152/physrev.00047.2005

[17] Broers JLV, Ramaekers FCS. The role of the nuclear lamina in cancer and apoptosis. Adv Exp Med Biol. 2014;773:27–48. doi: 10.1007/978-1-4899-8032-8_2 24563342 10.1007/978-1-4899-8032-8_2

[18] Broers JL, Raymond Y, Rot MK, Kuijpers H, Wagenaar SS, Ramaekers FC. Nuclear A-type lamins are differentially expressed in human lung cancer subtypes. Am J Pathol. 1993 7;143(1):211–20. 8391215 PMC1886958

[19] Kaufmann SH, Mabry M, Jasti R, Shaper JH. Differential expression of nuclear envelope lamins A and C in human lung cancer cell lines. Cancer Res. 1991 1 15;51(2):581–6. 1985776

[20] Belt EJT, Fijneman RJA, van den Berg EG, Bril H, Delis-van Diemen PM, Tijssen M, et al Loss of lamin A/C expression in stage II and III colon cancer is associated with disease recurrence. Eur J Cancer Oxf Engl 1990. 2011 8;47(12):1837–45. 10.1016/j.ejca.2011.04.025 21621406

[21] Willis ND, Wilson RG, Hutchison CJ. Lamin A: a putative colonic epithelial stem cell biomarker which identifies colorectal tumours with a more aggressive phenotype. Biochem Soc Trans. 2008 12;36(Pt 6):1350–3. doi: 10.1042/BST0361350 19021554 10.1042/BST0361350

[22] Willis ND, Cox TR, Rahman-Casañs SF, Smits K, Przyborski SA, van den Brandt P, et al Lamin A/C is a risk biomarker in colorectal cancer. PloS One. 2008;3(8):e2988 doi: 10.1371/journal.pone.0002988 18714339 10.1371/journal.pone.0002988 PMC2496895

[23] Moss SF, Krivosheyev V, de Souza A, Chin K, Gaetz HP, Chaudhary N, et al Decreased and aberrant nuclear lamin expression in gastrointestinal tract neoplasms. Gut. 1999 11;45(5):723–9. 10517909 10.1136/gut.45.5.723 PMC1727735

[24] Wazir U, Ahmed MH, Bridger JM, Harvey A, Jiang WG, Sharma AK, et al The clinicopathological significance of lamin A/C, lamin B1 and lamin B receptor mRNA expression in human breast cancer. Cell Mol Biol Lett. 2013 12;18(4):595–611. doi: 10.2478/s11658-013-0109-9 24293108 10.2478/s11658-013-0109-9 PMC6275779

[25] Coradeghini R, Barboro P, Rubagotti A, Boccardo F, Parodi S, Carmignani G, et al Differential expression of nuclear lamins in normal and cancerous prostate tissues. Oncol Rep. 2006 3;15(3):609–13. 16465420

[26] Saarinen I, Mirtti T, Seikkula H, Boström PJ, Taimen P. Differential Predictive Roles of A- and B-Type Nuclear Lamins in Prostate Cancer Progression. PloS One. 2015;10(10):e0140671 doi: 10.1371/journal.pone.0140671 26469707 10.1371/journal.pone.0140671 PMC4607298

[27] Machiels BM, Ramaekers FC, Kuijpers HJ, Groenewoud JS, Oosterhuis JW, Looijenga LH. Nuclear lamin expression in normal testis and testicular germ cell tumours of adolescents and adults. J Pathol. 1997 6;182(2):197–204. doi: 10.1002/(SICI)1096-9896(199706)182:2<197::AID-PATH823>3.0.CO;2-P 9274531 10.1002/(SICI)1096-9896(199706)182:2<197::AID-PATH823>3.0.CO;2-P

[28] Oguchi M, Sagara J, Matsumoto K, Saida T, Taniguchi S. Expression of lamins depends on epidermal differentiation and transformation. Br J Dermatol. 2002 11;147(5):853–8. 12410693 10.1046/j.1365-2133.2002.04948.x

[29] Venables RS, McLean S, Luny D, Moteleb E, Morley S, Quinlan RA, et al Expression of individual lamins in basal cell carcinomas of the skin. Br J Cancer. 2001 2;84(4):512–9. doi: 10.1054/bjoc.2000.1632 11207047 10.1054/bjoc.2000.1632 PMC2363768

[30] Capo-Chichi CD, Aguida B, Chabi NW, Cai QK, Offrin G, Agossou VK, et al Lamin A/C deficiency is an independent risk factor for cervical cancer. Cell Oncol Dordr. 2015 11 4; 10.1007/s13402-015-0252-6 PMC13001887 26537870

[31] Cicchillitti L, Corrado G, Carosi M, Dabrowska ME, Loria R, Falcioni R, et al Prognostic role of NF-YA splicing isoforms and Lamin A status in low grade endometrial cancer. Oncotarget. 2017 1 31;8(5):7935–45. doi: 10.18632/oncotarget.13854 27974701 10.18632/oncotarget.13854 PMC5352372

[32] Gong G, Chen P, Li L, Tan H, Zhou J, Zhou Y, et al Loss of lamin A but not lamin C expression in epithelial ovarian cancer cells is associated with metastasis and poor prognosis. Pathol Res Pract. 2015 2;211(2):175–82. doi: 10.1016/j.prp.2014.11.008 25499720 10.1016/j.prp.2014.11.008

[33] Wang Y, Wu R, Cho KR, Thomas DG, Gossner G, Liu JR, et al Differential protein mapping of ovarian serous adenocarcinomas: identification of potential markers for distinct tumor stage. J Proteome Res. 2009 3;8(3):1452–63. doi: 10.1021/pr800820z 19159301 10.1021/pr800820z PMC2693455

[34] World Health Organization Classification of Tumours. Pathology and Genetics of Tumours of the Lung, Pleura,Thymus and Heart. William D. Travis, Elizabeth Brambilla, H. Konrad Müller-Hermelink and Curtis C. Harris; 2004.

[35] Navarro CL, Cadiñanos J, De Sandre-Giovannoli A, Bernard R, Courrier S, Boccaccio I, et al Loss of ZMPSTE24 (FACE-1) causes autosomal recessive restrictive dermopathy and accumulation of Lamin A precursors. Hum Mol Genet. 2005 6 1;14(11):1503–13. doi: 10.1093/hmg/ddi159 15843403 10.1093/hmg/ddi159

[36] Estañol JM, Agell N, Bachs O. Nuclear protein patterns in normal T-lymphocytes and lymphoblastoid cells. Cancer Res. 1997 1 1;57(1):55–61. 8988041

[37] Perrin S, Cremer J, Faucher O, Reynes J, Dellamonica P, Micallef J, et al HIV protease inhibitors do not cause the accumulation of prelamin A in PBMCs from patients receiving first line therapy: the ANRS EP45 “aging” study. PloS One. 2012;7(12):e53035 doi: 10.1371/journal.pone.0053035 23285253 10.1371/journal.pone.0053035 PMC3532351

[38] Jung H-J, Coffinier C, Choe Y, Beigneux AP, Davies BSJ, Yang SH, et al Regulation of prelamin A but not lamin C by miR-9, a brain-specific microRNA. Proc Natl Acad Sci U S A. 2012 2 14;109(7):E423–31. doi: 10.1073/pnas.1111780109 22308344 10.1073/pnas.1111780109 PMC3289373

[39] Nissan X, Blondel S, Navarro C, Maury Y, Denis C, Girard M, et al Unique preservation of neural cells in Hutchinson- Gilford progeria syndrome is due to the expression of the neural-specific miR-9 microRNA. Cell Rep. 2012 7 26;2(1):1–9. doi: 10.1016/j.celrep.2012.05.015 22840390 10.1016/j.celrep.2012.05.015

[40] Lopez-Mejia IC, Vautrot V, De Toledo M, Behm-Ansmant I, Bourgeois CF, Navarro CL, et al A conserved splicing mechanism of the LMNA gene controls premature aging. Hum Mol Genet. 2011 12 1;20(23):4540–55. doi: 10.1093/hmg/ddr385 21875900 10.1093/hmg/ddr385

[41] Scaffidi P, Misteli T. Lamin A-dependent nuclear defects in human aging. Science. 2006 5 19;312(5776):1059–63. doi: 10.1126/science.1127168 16645051 10.1126/science.1127168 PMC1855250

[42] Das S, Krainer AR. Emerging functions of SRSF1, splicing factor and oncoprotein, in RNA metabolism and cancer. Mol Cancer Res MCR. 2014 9;12(9):1195–204. doi: 10.1158/1541-7786.MCR-14-0131 24807918 10.1158/1541-7786.MCR-14-0131 PMC4163531

[43] Gout S, Brambilla E, Boudria A, Drissi R, Lantuejoul S, Gazzeri S, et al Abnormal expression of the pre-mRNA splicing regulators SRSF1, SRSF2, SRPK1 and SRPK2 in non small cell lung carcinoma. PloS One. 2012;7(10):e46539 doi: 10.1371/journal.pone.0046539 23071587 10.1371/journal.pone.0046539 PMC3468597

[44] Tang Y, Chen Y, Jiang H, Nie D. Promotion of tumor development in prostate cancer by progerin. Cancer Cell Int. 2010;10:47 doi: 10.1186/1475-2867-10-47 21106101 10.1186/1475-2867-10-47 PMC3003644

[45] Okamoto S, Okamoto A, Nikaido T, Saito M, Takao M, Yanaihara N, et al Mesenchymal to epithelial transition in the human ovarian surface epithelium focusing on inclusion cysts. Oncol Rep. 2009 5;21(5):1209–14. 19360296 10.3892/or_00000343

[46] Zhai X, Zhu H, Wang W, Zhang S, Zhang Y, Mao G. Abnormal expression of EMT-related proteins, S100A4, vimentin and E-cadherin, is correlated with clinicopathological features and prognosis in HCC. Med Oncol Northwood Lond Engl. 2014 6;31(6):970. 10.1007/s12032-014-0970-z 24781336

[47] Liao G, Wang M, Ou Y, Zhao Y. IGF-1-induced epithelial-mesenchymal transition in MCF-7 cells is mediated by MUC1. Cell Signal. 2014 10;26(10):2131–7. doi: 10.1016/j.cellsig.2014.06.004 24971507 10.1016/j.cellsig.2014.06.004

[48] Nath S, Mukherjee P. MUC1: a multifaceted oncoprotein with a key role in cancer progression. Trends Mol Med. 2014 6;20(6):332–42. doi: 10.1016/j.molmed.2014.02.007 24667139 10.1016/j.molmed.2014.02.007 PMC5500204

[49] Xu T, Liu X, Han L, Shen H, Liu L, Shu Y. Up-regulation of miR-9 expression as a poor prognostic biomarker in patients with non-small cell lung cancer. Clin Transl Oncol Off Publ Fed Span Oncol Soc Natl Cancer Inst Mex. 2014 5;16(5):469–75. 10.1007/s12094-013-1106-1 24019037

[50] Gutschner T, Hämmerle M, Diederichs S. MALAT1—a paradigm for long noncoding RNA function in cancer. J Mol Med Berl Ger. 2013 7;91(7):791–801. 10.1007/s00109-013-1028-y 23529762

[51] Ji P, Diederichs S, Wang W, Böing S, Metzger R, Schneider PM, et al MALAT-1, a novel noncoding RNA, and thymosin beta4 predict metastasis and survival in early-stage non-small cell lung cancer. Oncogene. 2003 9 11;22(39):8031–41. doi: 10.1038/sj.onc.1206928 12970751 10.1038/sj.onc.1206928

[52] Schmidt LH, Spieker T, Koschmieder S, Schäffers S, Humberg J, Jungen D, et al The long noncoding MALAT-1 RNA indicates a poor prognosis in non-small cell lung cancer and induces migration and tumor growth. J Thorac Oncol Off Publ Int Assoc Study Lung Cancer. 2011 12;6(12):1984–92. 10.1097/JTO.0b013e3182307eac 22088988

[53] Shen L, Chen L, Wang Y, Jiang X, Xia H, Zhuang Z. Long noncoding RNA MALAT1 promotes brain metastasis by inducing epithelial-mesenchymal transition in lung cancer. J Neurooncol. 2014 9 14; 10.1007/s11060-014-1613-0 25217850

[54] Leucci E, Patella F, Waage J, Holmstrøm K, Lindow M, Porse B, et al microRNA-9 targets the long non-coding RNA MALAT1 for degradation in the nucleus. Sci Rep. 2013;3:2535 doi: 10.1038/srep02535 23985560 10.1038/srep02535 PMC3756333

[55] Lin R, Roychowdhury-Saha M, Black C, Watt AT, Marcusson EG, Freier SM, et al Control of RNA processing by a large non-coding RNA over-expressed in carcinomas. FEBS Lett. 2011 2 18;585(4):671–6. doi: 10.1016/j.febslet.2011.01.030 21266177 10.1016/j.febslet.2011.01.030 PMC3065235

[56] Tripathi V, Ellis JD, Shen Z, Song DY, Pan Q, Watt AT, et al The nuclear-retained noncoding RNA MALAT1 regulates alternative splicing by modulating SR splicing factor phosphorylation. Mol Cell. 2010 9 24;39(6):925–38. doi: 10.1016/j.molcel.2010.08.011 20797886 10.1016/j.molcel.2010.08.011 PMC4158944

[57] Weber DG, Johnen G, Casjens S, Bryk O, Pesch B, Jöckel K-H, et al Evaluation of long noncoding RNA MALAT1 as a candidate blood-based biomarker for the diagnosis of non-small cell lung cancer. BMC Res Notes. 2013;6:518 doi: 10.1186/1756-0500-6-518 24313945 10.1186/1756-0500-6-518 PMC4029199

[58] Lee DS, Kang JH, Lee CG, Kim SJ, Choi YJ, Lee KY, et al Predicting Survival in Patients with Advanced Non-squamous Non-small Cell Lung Cancer: Validating the Extent of Metastasis. Cancer Res Treat Off J Korean Cancer Assoc. 2013 6;45(2):95–102. 10.4143/crt.2013.45.2.95 PMC3710968 23864842

[59] Lee DS, Kim YS, Kay CS, Kim SH, Yeo CD, Kim JW, et al Distinctive Patterns of Initially Presenting Metastases and Clinical Outcomes According to the Histological Subtypes in Stage IV Non-Small Cell Lung Cancer. Medicine (Baltimore). 2016 2;95(6):e2795. 26871841 10.1097/MD.0000000000002795 PMC4753937

[60] Kuchuk M, Kuchuk I, Sabri E, Hutton B, Clemons M, Wheatley-Price P. The incidence and clinical impact of bone metastases in non-small cell lung cancer. Lung Cancer Amst Neth. 2015 8;89(2):197–202. 10.1016/j.lungcan.2015.04.007 26003503

[61] Heller G, Weinzierl M, Noll C, Babinsky V, Ziegler B, Altenberger C, et al Genome-wide miRNA expression profiling identifies miR-9-3 and miR-193a as targets for DNA methylation in non-small cell lung cancers. Clin Cancer Res Off J Am Assoc Cancer Res. 2012 3 15;18(6):1619–29. 10.1158/1078-0432.CCR-11-2450 22282464

[62] Lujambio A, Calin GA, Villanueva A, Ropero S, Sánchez-Céspedes M, Blanco D, et al A microRNA DNA methylation signature for human cancer metastasis. Proc Natl Acad Sci U S A. 2008 9 9;105(36):13556–61. doi: 10.1073/pnas.0803055105 18768788 10.1073/pnas.0803055105 PMC2528872

[63] Muraoka T, Soh J, Toyooka S, Maki Y, Shien K, Furukawa M, et al Impact of aberrant methylation of microRNA-9 family members on non-small cell lung cancers. Mol Clin Oncol. 2013 1;1(1):185–9. doi: 10.3892/mco.2012.18 24649145 10.3892/mco.2012.18 PMC3956236

[64] Simmons CP, Koinis F, Fallon MT, Fearon KC, Bowden J, Solheim TS, et al Prognosis in advanced lung cancer—A prospective study examining key clinicopathological factors. Lung Cancer Amst Neth. 2015 6;88(3):304–9. 10.1016/j.lungcan.2015.03.020 25870155

[65] Azzoli CG, Temin S, Giaccone G. 2011 Focused Update of 2009 American Society of Clinical Oncology Clinical Practice Guideline Update on Chemotherapy for Stage IV Non–Small-Cell Lung Cancer. J Oncol Pract. 2012 1;8(1):63–6. doi: 10.1200/JOP.2011.000374 22548014 10.1200/JOP.2011.000374 PMC3266319

[66] Tabchi S, Kassouf E, Florescu M, Tehfe M, Blais N. Factors influencing treatment selection and survival in advanced lung cancer. Curr Oncol. 2017 4 27;24(2):115. 10.3747/co.24.3355 PMC5407874 28490934

