# Supplementary figures and images for: Low lamin A expression in lung adenocarcinoma cells from pleural effusions is a pejorative factor associated with high number of metastatic sites and poor Performance status
Source: PLoS One. 2017 Aug 14;12(8):e0183136. doi: 10.1371/journal.pone.0183136 (PMC5555706; doi:10.1371/journal.pone.0183136)

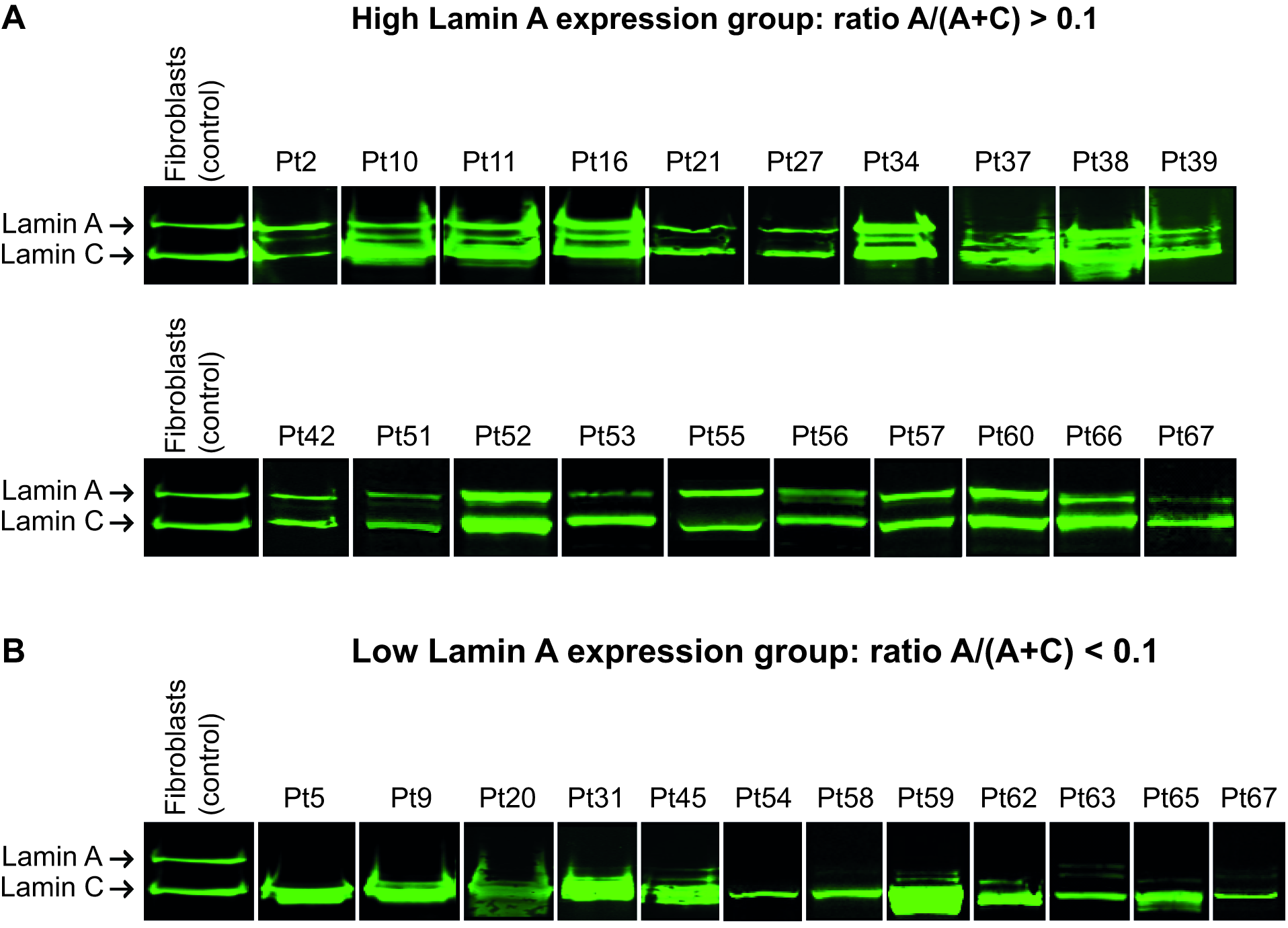

Supplement: S1 Fig — Representative results from various experiments using total protein extracts of dermal fibroblasts (control) and extracts of cells contained in metastatic pleural effusions from the 32 patients (Pt). Patients were separated according to the ratio A/(A+C). Patients with a ratio higher than 0.1 belonged to the “High lamin A expression group” whereas patients with a ratio under 0.1 were classified in the “Low lamin A expression group”. (TIF) [file pone.0183136.s001.tif]

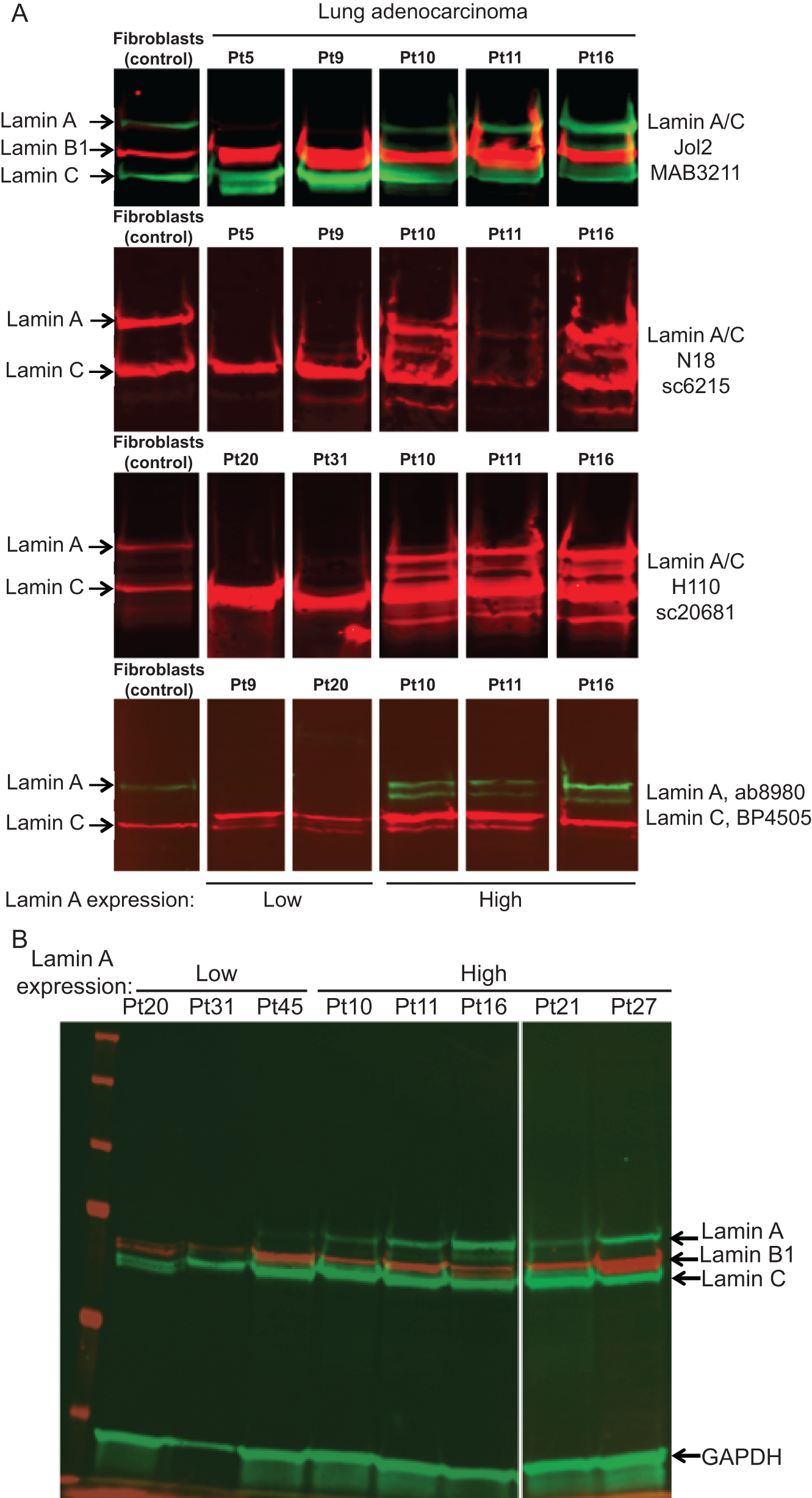

Supplement: S2 Fig — (A) Representative results of western blot analysis of nuclear extracts from patients (Pt) 5, 9, 10, 11, 16, 20 and 31 and from control dermal fibroblasts using a mouse anti-lamin A/C antibody (Jol2, MAB3211), a goat-anti-lamin A/C antibody (N18, sc6215), a rabbit-anti-lamin A/C antibody (H110, sc20681), anti-lamin A antibody (ab8980) and anti-lamin C antibody (BP4505). (B) Representative results of western blot analysis of total protein extracts from patients (Pt) 20, 31, 45 (low lamin A group) and Pt 10, 11, 16, 21 and 27 (from high lamin A group) using a mouse anti-lamin A/C antibody (Jol2). (TIF) [file pone.0183136.s002.tif]
